# Quantitative analysis of taxane drug target engagement of microtubules in circulating tumor cells from metastatic castration resistant prostate cancer patients treated with CRXL301, a nanoparticle of docetaxel

**DOI:** 10.20517/cdr.2019.116

**Published:** 2020-04-17

**Authors:** Eiman Mukhtar, Daniel Worroll, Giuseppe Galletti, Shelly Schuster, Sarina A. Piha-Paul, Paraskevi Giannakakou

**Affiliations:** ^1^Department of Medicine, Hematology/Oncology, Weill Cornell Medicine, New York, NY 10065, USA.; ^2^Sandra and Edward Meyer Cancer Center, Department of Medicine, Weill Cornell Medicine, New York, NY 10065, USA.; ^3^NewLink Genetics Corporation, Ames, IA 50010, USA.; ^4^The University of Texas, MD Anderson Cancer Center, Houston, TX 77030, USA.

**Keywords:** Liquid biopsy, circulating tumor cells, castration-resistant prostate cancer, microtubules, taxanes, biomarkers

## Abstract

**Aim:** We reviewed the radiographic response of three patients with metastatic castration-resistant prostate cancer treated with CRXL301, a docetaxel nanoparticle. For these three patients, we isolated and analyzed circulating tumor cells (CTCs) to explore microtubule (MT) drug-target engagement (MT-DTE) as a biomarker of response to treatment. MT-DTE was based on a quantitative assessment of the MT cytoskeleton in CTCs from pre- and post-treatment patient samples as a potential read-out of CRXL301 activity.

**Methods:** We isolated CTCs using negative CD45^+^ depletion and subjected them to multiplex confocal microscopy using our established protocol. CTCs were identified as CD45^-^/CK^+^/DAPI^+^ cells and MT-DTE was determined using our developed imaging algorithm. We quantified MT bundling in CTCs across multiple time points, from baseline to on-treatment to disease progression. Here, we describe the longitudinal analysis of MT-DTE in CTCs from patients treated with CRXL301 and its correlation with response to treatment.

**Results:** We collected CTCs at seven time points from three metastatic castration-resistant prostate cancer patients. Clinical response was evaluated by Response Evaluation Criteria in Solid Tumors (RECIST) v.1.1 criteria in those patients with measurable disease. Of the three patients enrolled, one experienced partial response (-50%) to CRXL301 and two patients were unevaluable given bone only disease. Notably, however, these two patients showed stable disease clinically based on bone scans. MT-DTE across all time points revealed that, early time points within four and 24 h of drug administration exhibited the highest levels of drug engagement (MT-DTE) as compared to baseline. However, these early time points did not correlate with clinical response. We observed that the CTCs collected one week after the first or second dose of CRXL301 treatment in the responding patient had numerically higher levels of MT-DTE as compared to the other two patients.

**Conclusion:** Taxane on-target activity can be detected and analyzed quantitatively in CTCs by tubulin immunofluorescence. Early time points, within 24 h of drug administration, showed high levels of DTE but did not correlate with clinical response. MT-DTE in CTCs collected after one week on treatment correlated best with treatment response. The clinical utility of the 1-week CTC DTE should be tested and validated in future clinical trials involving taxanes.

## Introduction

Microtubule-targeting drugs represent one of the most clinically active chemotherapy drugs for the treatment of solid tumors^[1]^. Particularly for prostate cancer, taxanes (docetaxel and cabazitaxel) are the only chemotherapy shown to prolong survival in metastatic castration-resistant prostate cancer (mCRPC) patients, and are now considered standard of treatment^[2-5]^. Despite their clinical success, unfortunately not all patients benefit from taxane treatment, while the molecular basis for taxane resistance in CRPC remains unknown. Currently, there is no biomarker indicative of taxane clinical activity that can be used to discriminate responding *vs*. non-responding patients early on during their treatment.

At the molecular level, taxanes bind to the β-tubulin subunit of microtubules (MT), which are tubulin polymers involved in many biological functions, such as intracellular trafficking of molecules and organelles, and cell mitosis^[6-8]^. Following taxane binding to their primary cellular target, tubulin, MT stabilization and increase in MT polymer mass occur. This is indicative of effective (drug-target engagement, DTE). This MT-DTE is visually evidenced by reorganization of the MT network into distinct MT bundles. Impaired MT-DTE has been associated with taxane resistance in multiple preclinical models^[9,10]^. However, no clinical studies assessing MT-DTE on tumor samples have been reported yet, and the role of MT-DTE as biomarker of clinical response to taxane chemotherapy is still unclear.

Despite their clinical success, the administration of taxanes is a long, and complex procedure, which requires the use of highly allergenic amphiphilic solution (i.e., Cremophor) and extensive steroid prophylaxis due to their hydrophobic structure^[11]^.

The emergence of nanotechnology, particularly nanoparticle-drug conjugates, has improved docetaxel delivery, because it offers the advantages of unique size, improved drug solubility, passive targeting by enhanced permeability and retention effect, and more controlled drug release to the tumor^[11-15]^.

Currently, the molecular characterization of tumor tissue requires a tumor biopsy. However, biopsies are invasive and risky procedures with significant limitations, including biopsy-site bias due to tumor heterogeneity, and challenges associated with repeat biopsies on the same patient^[16,17]^. Molecular analyses of circulating tumor cells (CTCs), isolated from the peripheral blood of cancer patients with a simple blood draw, have emerged as alternative validated sources of tumor cells. Although first described in 1869^[18]^, only recently CTCs have shown their clinical relevance, after clinical studies utilized CTCs as source of tumor material to analyze biomarkers of response to treatment in cancer patients^[19,20]^.

In the present study (CRXL301-101; ClinicalTrial.gov identifier: NCT02380677), we isolated CTCs from the peripheral blood of CRPC patient receiving the investigational drug CRXL301, a payload docetaxel covalently conjugated to a cyclodextrin-polyethylene glycol co-polymer. This is designed to concentrate in tumors, due to the unique characteristics of tumor’s vasculature, and slowly release docetaxel inside tumor cells. CTCs were used to explore the utility of using immunofluorescence to quantitatively assess the integrity of MT cytoskeleton from on-treatment blood samples compared to baseline, as a potential read-out of CRXL301 clinical activity. CTCs were quantitatively analyzed at the single-cell level for MT-DTE to determine if this measure correlated with treatment response to nanoparticle delivery of docetaxel on an individual basis.

## Methods

### Patient characteristics and study design

Patients with mCRPC were enrolled in the phase 2a study CRXL301-101 to receive CRXL301, payload docetaxel covalently conjugated to a cyclodextrin-polyethylene glycol co-polymer that is 10-30 nm in diameter. All subjects received the same dose and schedule of CRXL301. Patients had previously been treated with AR targeted therapies (e.g., abiraterone and/or Enzalutamide) but were required not to have received any prior taxane-based chemotherapy. Patients were also required to have adequate organ function and an Eastern Cooperative Oncology Group performance score of 0 to 1^[21]^.

The study was approved by the institutional review board at MD Anderson Cancer Center where these three patients were treated. Patients provided written informed consent before participation. NewLink Genetics granted the permission to release the clinical data on these three prostate cancer patients whose samples were analyzed.

Patients were followed for safety, tolerability and preliminary tumor efficacy. RECIST v.1.1 was used for those patients with measureable disease. Patients continued treatment until they experienced progression of disease or unacceptable toxicity or patient/physician decision to withdraw from study.

### Blood collection and CTC enrichment

Ten mL of peripheral blood was collected in EDTA tubes (BD Vacutainer® Franklin Lakes, NJ) from three mCRPC patients before initiation of CRXL301 chemotherapy (baseline; cycle 1 day 1; C1D1). Additionally, 10 mL of peripheral blood was also collected at the following time points: C1D1 plus four hours, C1D2, C1D8, C2D1, C2D1 plus four hours, and C2D8.

Blood samples were shipped at ambient temperature on same day of collection and were processed within 24 h from blood draw. Samples were processed as previously described^[22]^.

Briefly, CTCs were enriched by negative depletion of CD45^+^ cells (peripheral mononuclear cells, PBMCs) by using the RosetteSep^TM^ Human CD45 Depletion Cocktail (Stemcell Technologies, Cambridge, MA); the enriched CTCs were subsequently cytospun onto coverslips and processed for immunofluorescence staining.

### Immunofluorescence

Enriched CTC cell preparations were plated on coverslips and fixed with pre-warmed (37 °C) 2% formaldehyde (Sigma-Aldrich, St. Louis, MO) in 1× PHEM buffer (60 mM PIPES, 25 mM HEPES, 10 mM EGTA, 2 mM MgCl_2_) for 15 min and blocked overnight in 10% Normal Goat Serum (Jackson ImmunoResearch, West Grove, PA) plus 6% Bovine Serum Albumin (BSA) in PBS (Corning, Tewksbury, MA).

Cells were subsequently immunostained for: (1) the leukocyte marker CD45 using an anti-CD45 antibody directly conjugated with QDot-800 (Invitrogen, Waltham, MA Catalog # Q10156); (2) the epithelial marker cytokeratin, using an anti-pan-cytokeratin mouse monoclonal antibody recognizing human cytokeratins 4, 5, 6, 8, 10, 13, and 18 (BioLegend, San Diego, CA catalog # 628602) conjugated to CF594 using Biotium Mix-n- Stain antibody labeling kit (catalog # 92236); (3) tubulin (Novus rat anti-tyrosinated tubulin, Catalog # NB600-506), followed by goat anti-rat IgG secondary antibody, AlexaFluor 647 (Thermo Fisher Scientific, catalog # A21247); (4) androgen receptor AR [Rabbit anti-AR (*N*-terminal) catalog # ab3510], and goat anti-rabbit IgG secondary antibody, AlexaFluor 488 (Catalog # A11034); and (5) DAPI for nuclear counterstaining (Invitrogen). Coverslips were mounted on a glass slide by using Mowiol mounting media (Electronic Microscopy Sciences, Hatfield, PA).

### CTC identification and high-resolution imaging

After immunostaining, CTCs were identified according to the standard definition as nucleated cells (DAPI^+^), positive for cytokeratin (CK) as a marker of epithelial origin, and negative for CD45, a marker of leukocytes.

Multiplex confocal microscopy was performed as previously described^[22]^. Briefly, low-resolution (10× magnification) tile scans of coverslips were obtained. Nucleated cells were quantitatively analyzed for morphological features such as size and shape, as well as fluorescence intensities of CD45, CK, and DAPI to identify putative CTCs with the established DAPI^+^/CK^+^/CD45^−^ staining phenotype. Putative CTCs were also required to have positive tubulin staining for downstream analysis as well as positive AR staining indicative of prostate cancer origin. Cells that fit these criteria were subjected to high-resolution (63× magnification) multiplex confocal microscopy. Approximately 20-40 individual z-slices at 0.24 μm per z-plane were recorded for each cell. Imaging conditions (exposure time and laser intensity) were set to avoid saturation and were kept constant for all CTCs imaged across all patients. Putative cells subjected to high-resolution imaging were confirmed as CTCs through manual, operator-dependent image validation prior to downstream, quantitative MT-DTE analysis. CTCs were also required to have positive tubulin staining for downstream analysis as well as positive AR staining indicative of prostate cancer origin.

### Quantitative analysis of CRXL301 DTE

Three-dimensional reconstructions of CTCs were generated following high- resolution image acquisition by confocal microscopy. CTCs were rank-ordered from high to low CK^+^ staining intensity. Up to 20 confirmed CTCs at each time point per patient were then subjected to analysis of MT-DTE.

For this purpose, we developed a semi-automated scoring algorithm to quantify MT-DTE. This methodology avoids qualitative, operator-based assessment of MT bundling, and utilizes the fluorescent pixel intensity distribution for each image. All image analyses were performed in the ImageJ software environment^[23]^.

Briefly, to quantify the MT-DTE for each single CTC the maximum intensity projection for the tubulin channel was exported as a single, 8-bit TIFF. The tubulin maximum intensity projection was initially thresholded to define a region of interest, which was used for fluorescence intensity quantitation. Threshold calculations were performed for each individual image based on the dynamic range of pixel distribution. We first calculated the maximum pixel intensity within the image and set this number as the upper bound of the threshold. The lower bound of the threshold was calculated as 75% of the maximum pixel value. This generated a threshold corresponding to pixels with tubulin fluorescence intensities within the top 25 percentile of fluorescent intensity values.

Using the threshold region of interest, MT-DTE was calculated as the integrated density (mean fluorescence intensity multiplied by the area of the threshold region of interest). MT-DTE was calculated for each CTC, at each time point, for each patient.

## Results

### CTC enrichment from mCRPC patient and image acquisition

**Figure 1:**
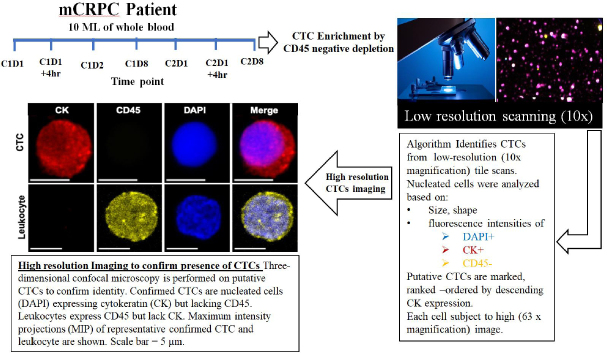
Workflow of CTC isolation from CRPC patient peripheral blood and multiplex confocal microscopy imaging for drug-target engagement quantification. CTC: circulating tumor cell; mCRPC: metastatic castration-resistant prostate cancer

We collected peripheral blood from three mCRPC patients enrolled in a phase 2a trial with CRXL301, a nanoparticle conjugate of docetaxel. All three patients received at least two cycles of CRXL301. Blood samples were prospectively collected from the three patients at baseline, before the administration of the first cycle of treatment (C1D1), and at different time points on treatment [Figure 1].

The goal of this observational study was to determine the effect of the investigational drug on its target, the microtubule cytoskeleton, in patient CTCs and correlate with response to treatment. We developed a quantitative algorithm to determine the drug-induced MT stabilization, hereafter MT-DTE. To do that we used our established pipeline of CTC enrichment via CD45 negative depletion, followed by a high-throughput imaging algorithm which identifies putative CTCs based on low- resolution scanning using established criteria of DAPI^+^/CK^+^/CD45^-^ immunostaining. Putative CTCs were then subjected to high-resolution confocal microscopy to confirm CTC identity [Figure 1]. Furthermore, to confirm prostate cancer origin of our circulating cells, in addition to epithelial cell origin, we co-stained CTCs for AR using an *N*-terminal specific AR antibody [Supplementary Figure 1].

### Patient response to treatment

All patients received single agent CRXL301 for a minimum of two cycles. Patient response to treatment was evaluated according to RECIST. Among the three patients, we observed a differential response to treatment [Table 1]. According to RECIST Patient #1 achieved partial radiologic response with a 50% shrinkage in tumor size, while Patient #2 and Patient #3 benefitted only partially from the treatment, having stable disease according to bone scans [Table 1]. Additionally, the first patient was on the study for 7.3 months while the other two patients were only for 1.1 months (patient #2) and 3.8 months (patient #3). The differential clinical response to the investigational agent allowed us to evaluate as MT-DTE in CTCs as a potential biomarker of response to CRXL301.

**Table 1:** Patient clinical characteristics and number of CTCs analyzed per time point.

	Time point	Number of analyzed CTCs	Mean MT-DTE (SD)	Time on study (months)	Best PSA response on treatment	Best RECIST v.1.1 response
Patient 1	C1D1	3	0.19 (0.16)	7.27	-44%	Partial response (-50%)
	C1D1 + 4 h	1	0.004			
	C1D2	2	0.34 (0.26)			
	C1D8	6	1.0 (0.85)			
	C2D1	2	0.17 (0.15)			
	C2D1 + 4 h	17	8.3 (7.8)			
	C2D8	11	0.45 (0.32)			
Patient 2	C1D1	3	0.33 (0.44)	1.15	-35%	Stable disease (not measureable)
	C1D1 + 4 h	20	16.39 (14.43)			
	C1D2	5	22.87 (18.78)			
	C1D8	2	0.44 (0.081)			
	C2D1	10	27.21 (26.79)			
	C2D1 + 4 h	17	20.04 (23.58)			
	C2D8	Sample not received	N/A			
Patient 3	C1D1	5	14.80 (18.8)	3.8	43%	Stable disease (not measureable)
	C1D1 + 4 h	14	5.07 (16.92)			
	C1D2	Sample not received	N/A			
	C1D8	Sample not received	N/A			
	C2D1	12	46.64 (25.67)			
	C2D1 + 4 h	17	14.47 (12.14)			
	C2D8	2	0.1185 (0.040)			

Time points for CTC enrichment are shown for each patient. Column 2 displays the number of confirmed CTCs analyzed for each time point, along with the mean MT-DTE score for (column three). Patient clinical characteristics such as time on study, best PSA response and clinical response by RECIST criteria are shown in the last three columns. SD: standard deviation; CTC: circulating tumor cell; MT-DTE: microtubule drug-target engagement; RECIST: response evaluation criteria in solid tumors; PSA: prostate specific antigen

### Quantitation of CRLX 301-induced MT-DTE in mCRPC patient CTCs

To quantify MT-DTE we analyzed a total of 149 confirmed CTCs from the three patients across all time points. Overall, Patient #1 exhibited lower CTC counts across all the time points (mean CTC count: 6; range: 1-17); conversely, the two patients who did not achieve an objective response showed numerically higher CTC counts (Patient #2 mean CTC: 9.5, range: 2-20; Patient #3 mean CTC: 10, range: 2-17). CTC counts, mean MT-DTE for all time points and patients’ clinical characteristics are shown in Table 1.

**Figure 2:**
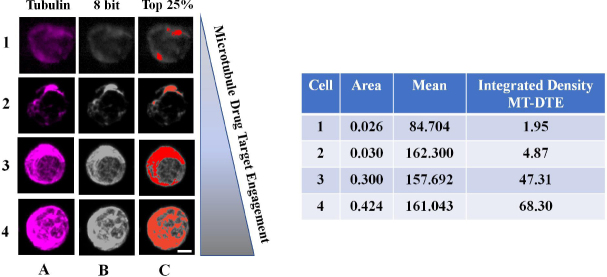
Workflow of quantitation of MT-DTE in representative images of mCRPC patient CTCs. Representative CTCs with different levels of DTE are shown, from least engaged (top row) to most engaged (bottom row). We quantified drug-induced MT-DTE using ImageJ as described in Methods. Tubulin maximum intensity projection is shown in column A, converted to the 8-bit TIFF image (column B). Column C shows visually the number of pixels corresponding to the top 25% of fluorescence intensity in each cell. Table shows resulting integrated density values (MT-DTE) for each single CTC. Scale bar = 20 μM. DTE: drug-target engagement; MT-DTE: microtubule drug-target engagement; mCRPC: metastatic castration-resistant prostate cancer; CTCs: circulating tumor cells

To quantitatively measure MT-DTE, we used tubulin immunofluorescence and high-resolution confocal microscopy to generate three-dimensional reconstructions of MT structures and subjected them to quantitative analysis by ImageJ. MT-DTE was determined on a single cell basis by the mean integrated density of pixels in the top 25% of raw fluorescent intensity values. Representative images of CTCs with different levels of MT-DTE are shown in Figure 2.

CTCs with diffuse MT staining pattern and/or lack of discernible filamentous structures had lower MT-DTE. Signs of bundling including thick MT structures, disorganized MT network (distorted pattern throughout cell), and high MT fluorescence staining, had a higher MT-DTE [Figure 2]. For each patient the mean value of CTC MT-DTE was calculated at each time point and correlated with response to therapy.

At baseline (C1D1), the responding Patient #1, had numerically lower MT-DTE (mean: 0.190) compared to the baseline MT-DTE in the two patients with stable disease (Patients #2, Patient #3; means: 0.33, 14.80, respectively). In addition, four hours after treatment, Patient #1 had only one CTC detected, while Patients #2 and #3 had higher number of CTCs (*n* = 20 and 14, respectively) as well as numerically higher MT-DTE (Patient #1; mean: 0.004 compared to Patient #2, Patient #3; means: 16.39, 5.070, respectively) [Table 1].

We observed numerically higher levels of MT-DTE in CTCs collected within the first 24 h after drug administration (C1D2), compared to baseline in both Patients #1 and #2. For Patient #3, the C1D2 sample was not received. Considering that Patients #2 and #3 had stable disease whereas Patient #1 had a partial response to therapy, these early times points for MT-DTE at 4 and 24 h of treatment do not seem to discriminate the responding patient from the patients with stable [Table 1 and Supplementary Figure 2].

Interestingly, we observed that the responder, Patient #1, had consistently a numerically higher MT-DTE score one week after treatment administration (C1D8, C2D8) as compared to their respective baselines C1D1 and C2D1.

**Figure 3:**
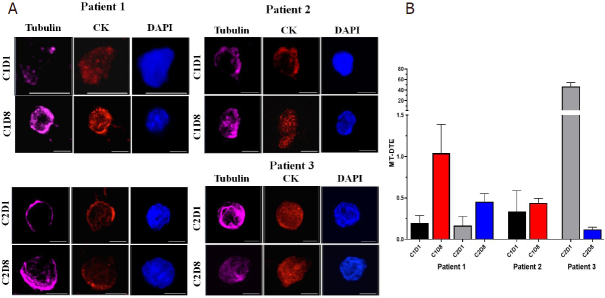
Representative images of mCRPC patient CTCs with quantitation of CRLX301-induced MT-DTE. A: single CTCs from patients 1-3 at different time points as indicated. CTCs are stained for tubulin (magenta), Pan-CK (red) and DAPI (blue) and imaged by high-resolution confocal microscopy. Maximum intensity projections are shown. Scale bar = 5 μM; B: graphical representation of mean MT-DTE (displayed as Mean ± SEM) at C1D1, C1D8, C2D1 and C2D8. mCRPC: metastatic castration-resistant prostate cancer; CTCs: circulating tumor cells; MT-DTE: microtubule drug-target engagement; CK: cytokeratin; DAPI: 4′, 6-diamidino-2-phenylindole

On the contrary, CTCs isolated from the patients with stable disease had either similar levels or a significant drop in MT-DTE a week after treatment administration (Patient #2; C1D8 *vs.* C1D1; means: 0.44 *vs.* 0.33) (Patient #3; C2D8 *vs.* C2D1; means: 0.119 *vs.* 46.64) [Figure 3 and Table 1].

Taken together, these data suggest that increases in MT-DTE one week after therapy with CRXL301 may be able to differentiate patients based on treatment response better than early time points within 24 h of treatment.

## Discussion

Taxanes (docetaxel and cabazitaxel) represent the most active class of chemotherapeutic drugs approved for the treatment of solid tumors to date. However, due to their toxicity profile and their hydrophobic structure, which compels extensive steroid prophylaxis, many attempts have been made to improve taxanes pharmacology. Docetaxel-conjugate nanoparticle, CRXL301, was designed to enhance drug delivery to tumor tissue up to 10 times more docetaxel into tumors than an equivalent milligram dose of commercially available docetaxel. This thus increases the activity and lowers the toxicity profile in patient^[24,25]^. At the cellular level, taxanes bind to and stabilize microtubules, inducing cell death by compromising their function during interphase and mitosis^[26]^. Therefore, microtubule stabilization (bundling) is the first sign of DTE^[1,27]^.

In our previous studies, we could not implement quantitative analysis of taxane-induced MT bundling in mCRPC patient CTCs, as MT integrity was not preserved in the CTCs isolated via the PSMA-based microfluidic device^[28]^. Instead, we quantified nuclear AR as a read-out of taxane clinical efficacy. In the current study, we used an antigen-agnostic method for CTC enrichment that did not disturb the MT cytoskeleton by mechanical forces or flow dynamics. The CTC cytoskeletal integrity allowed us to develop a quantitative algorithm for MT-DTE scoring based on tubulin immunofluorescence analysis and used it as potential read-out of CRXL301 clinical activity in mCRPC patients. We collected CTCs at seven time points from three mCRPC patients. MT-DTE was observed in all patients at early time points, within 24 h of drug administration [Table 1, Supplementary Figure 2]. However, it did not discriminate the responding patient from the patients with stable disease. In contrast, the higher MT-DTE in CTCs collected one week after the first or second dose of CRXL301, compared with their respective baseline samples, and was observed only in the responding patient. Taken together, these data suggest that early MT-DTE likely reflects initial drug binding but not sustained target engagement, while persistent MT-DTE after one week of treatment appears to correlate better with treatment response.

Despite the observational nature of these results and due to the limited sample size, our findings here are consistent with our previous report where we showed that a decrease in nuclear AR, likely as a result of MT stabilization^[29]^, in mCRPC patient CTCs one week after treatment initiation with docetaxel or cabazitaxel was significantly correlated with response to taxane chemotherapy^[28]^. However, MT-DTE was only qualitatively assessed in that study due to loss of cytoskeletal integrity after CTC enrichment. In the current study, we quantified MT-DTE and sought to correlate with AR subcellular localization in the three participating patients. We observed three distinct phenotypes of AR subcellular localization from the on-treatment CTC analysis, ranging from nuclear to cytoplasmic enrichment to downregulated. We did not observe a significant correlation between MT-DTE and cytoplasmic AR enrichment [Supplementary Figure 1], likely due to intra-patient heterogeneity in AR phenotypes together with the small sample size. In addition, we utilized a AR primary antibody which cannot discriminate between full-length AR and AR splice variants, which are constitutively nuclear and associated with taxane resistance^[30]^; this could in part explain the absence of correlation between MT engagement and AR sub cellular localization.

We also observed intra and inter patient heterogeneity in CTC MT-DTE analysis. We observed CTCs with no bundling from the on-treatment cohort, suggesting MT cytoskeleton response heterogeneity. This mixed response at CTC level could indicate clonal heterogeneity potentially due to inherent mechanisms of taxane resistance present in some but not all cells, such as tubulin mutations or expression of drug efflux pumps that could impair on target-drug engagement. Additional factors not yet studied in the clinical context, such as variability in cell biophysical properties (e.g., cell deformation), intracellular viscosity, and MT stiffness, could affect the formation of MT bundles in response to taxane treatment^[31]^. Better mechanistic understanding and the development of more sophisticated, multi-parametric computational approaches are required to develop a MT-bundling heterogeneity index, which could enable precision medicine strategies for patients receiving treatment with a microtubule stabilizing agent.

Regarding the inter-patient heterogeneity, we observed a pronounced MT-DTE at C1D1 in Patient #3, in the absence of prior taxane treatment, while there was no additional MT engagement following CRXL301 administration. This high MT bundling at baseline, may reflect aberrant MT stabilization by endogenous or secreted factors, or it may reflect dietary factors, such as the natural flavonoid, Fisetin, found in fruits and vegetables, which was shown to bind tubulin and stabilize microtubules with binding characteristics superior than paclitaxel^[32,33]^. Nevertheless, this observation warrants a bigger cohort to identify whether the presence of MT stabilization prior to taxane treatment may correlate with a lack of taxane drug efficacy.

Interestingly, a recent study reported on the clinical efficacy of BIND-014, a PSMA-directed docetaxel-containing nanoparticle, in metastatic CRPC patients. The study was performed in 42 patients and identified a significant association between a decline in PSMA-expressing CTCs preferentially and the clinical activity of the investigational agent^[34]^. Similar to our approach, this study assessed on target drug activity, which was limited to PSMA expression without taking into account MT changes, in response to docetaxel binding. Nevertheless, this approach is commendable and should be extended to all investigational drugs and their targets in patient CTCs. Such analyses can help select patient more likely to respond to treatment and provide mechanistic insight into the molecular basis of clinical response and resistance.

Overall, these data provide proof of principle that MT bundling induced by taxanes including their nanoparticle formulations can be detected in patient CTCs and that liquid biopsies at early, on treatment time points could be used to inform clinical decision making. Larger prospective clinical studies in solid tumors treated with taxane-based chemotherapy are warranted to validate these observations.

## Declarations

### Availability of data and materials

All data supporting our findings can be found within the manuscript and in the supplement data.

### Copyright

© The Author(s) 2022.
