# Supplementary figures and images for: Quantitative analysis of taxane drug target engagement of microtubules in circulating tumor cells from metastatic castration resistant prostate cancer patients treated with CRXL301, a nanoparticle of docetaxel
Source: Cancer Drug Resist. 2020 Apr 17;3(3):636–46. doi: 10.20517/cdr.2019.116 (PMC7556717; doi:10.20517/cdr.2019.116)

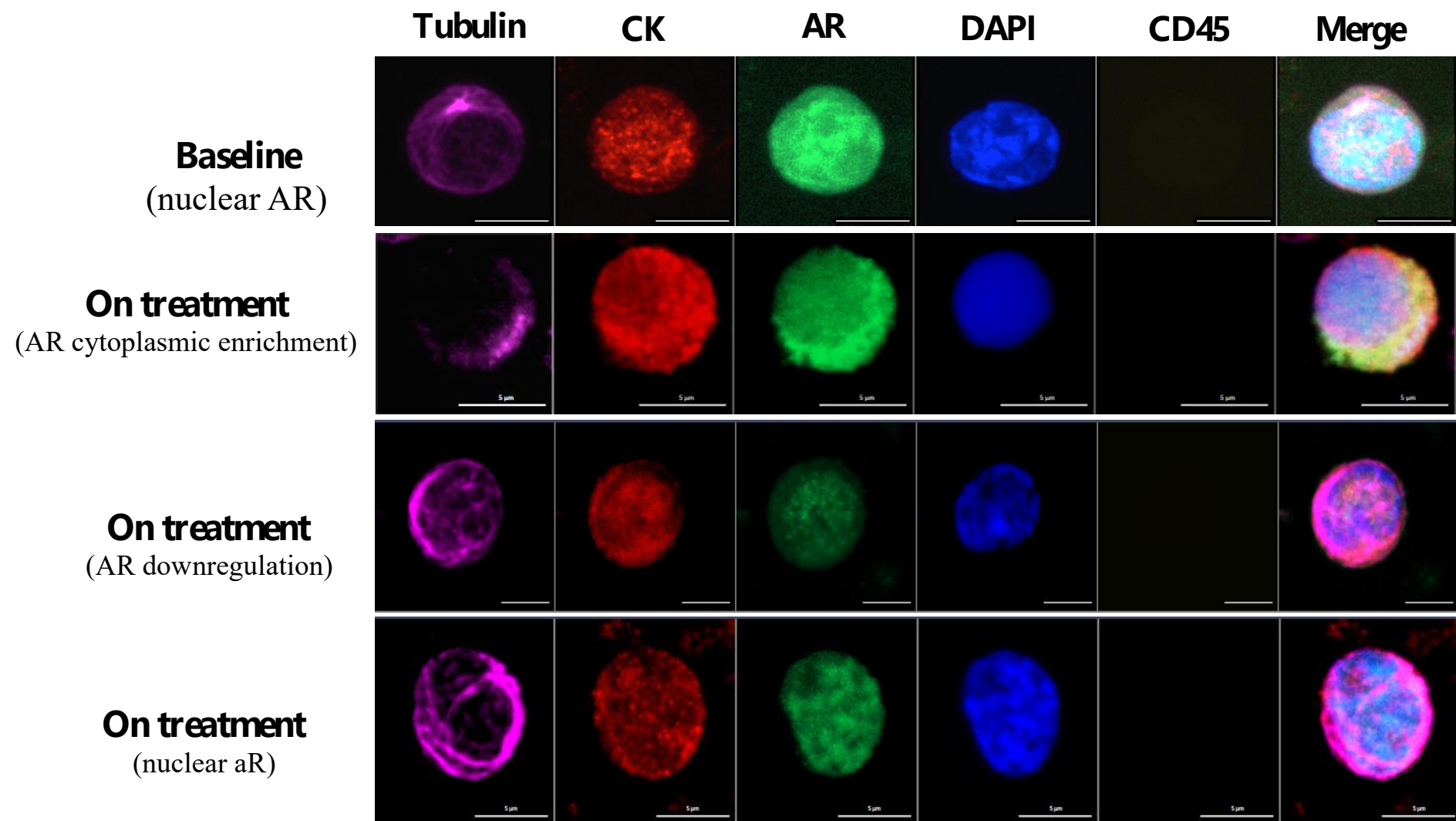

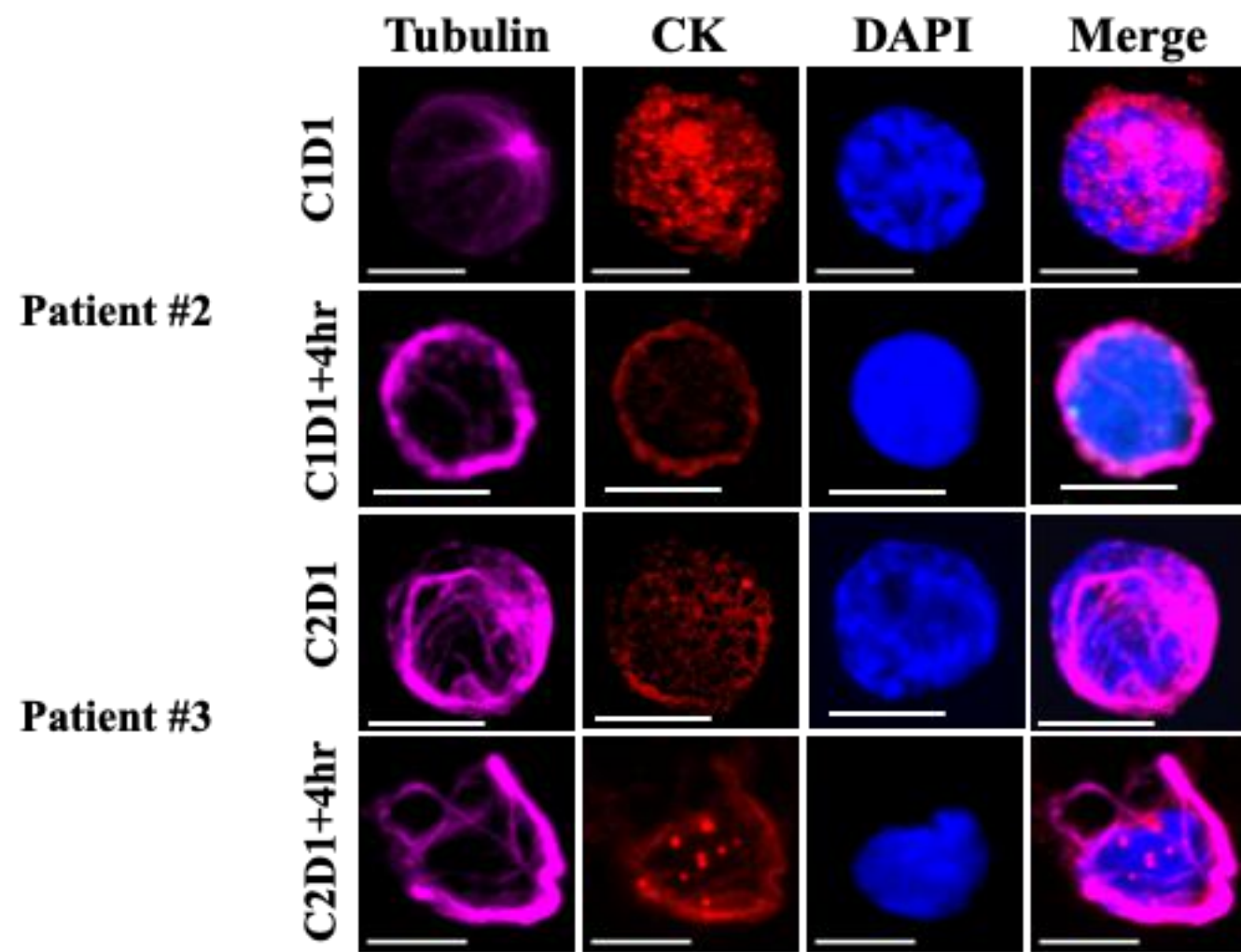

Supplement: Supplementary file 1 [file cdr-3-636-SupplementaryMaterials.pdf]
